# Thermodynamic Study on Biomimetic *Legionella gormanii* Bacterial Membranes

**DOI:** 10.3390/molecules29184367

**Published:** 2024-09-14

**Authors:** Katarzyna Pastuszak, Marta Palusińska-Szysz, Agnieszka Ewa Wiącek, Małgorzata Jurak

**Affiliations:** 1 Department of Interfacial Phenomena, Institute of Chemical Sciences, Faculty of Chemistry, Maria Curie-Skłodowska University, Maria Curie-Skłodowska Sq. 3, 20-031 Lublin, Poland; katarzyna.pastuszak2@mail.umcs.pl (K.P.); agnieszka.wiacek@mail.umcs.pl (A.E.W.); 2 Department of Genetics and Microbiology, Institute of Biological Sciences, Faculty of Biology and Biotechnology, Maria Curie-Skłodowska University, Akademicka 19, 20-033 Lublin, Poland; marta.palusinska-szysz@mail.umcs.pl

**Keywords:** *Legionella gormanii*, phospholipids, LL-37 peptide, Langmuir monolayer technique, model membranes, thermodynamic analysis, interactions

## Abstract

The presented studies were aimed at determining the interactions in model membranes (Langmuir monolayers) created of phospholipids (PL) isolated from *Legionella gormanii* bacteria cultured with (PL + choline) or without (PL − choline) choline and to describe the impact of an antimicrobial peptide, human cathelicidin LL-37, on PL’s monolayer behavior. The addition of choline to the growth medium influenced the mutual proportions of phospholipids extracted from *L. gormanii*. Four classes of phospholipids—phosphatidylcholine (PC), phosphatidylethanolamine (PE), phosphatidylglycerol (PG), cardiolipin (CL), and their mixtures—were used to register compression isotherms with or without the LL-37 peptide in the subphase. Based on them the excess area ($A_{e}$), excess ($\Delta G_{e}$), and total ($\Delta G_{m}$) Gibbs energy of mixing were determined. The thermodynamic analyses revealed that the PL − choline monolayer showed greater repulsive forces between molecules in comparison to the ideal system, while the PL + choline monolayer was characterized by greater attraction. The LL-37 peptide affected the strength of interactions between phospholipids’ molecules and reduced the monolayers stability. Accordingly, the changes in interactions in the model membranes allowed us to determine the difference in their susceptibility to the LL-37 peptide depending on the choline supplementation of bacterial culture.

## 1. Introduction

*Legionella* spp. are widely prevalent, colonizing various ecological niches with a preference for warm freshwater environments. The main epidemiological reservoirs for *Legionella* spp. are man-made structures, such as plumbing systems and air-conditioning units. These settings, especially in summer, harbor warm water pools supporting the co-culturing of *Legionella* spp. with their protozoan hosts. Once released from protozoan host cells, *Legionella* spp. can exist as free, motile planktonic bacteria capable of directly reinfecting new hosts or integrating into multispecies biofilm communities common within water systems. These biofilms are grazed by bacterivorous protozoa, facilitating interactions between protozoa and bacteria. A direct consequence of the ecology of *Legionella* spp. as protozoan parasites is their ability to infect human cells. Bacteria are transmitted from the environment to humans by inhaling *Legionella*-laden aerosols. In humans, *Legionella* spp. can lead to a potentially fatal pneumonia known as Legionnaires’ disease and a milder illness referred to as Pontiac fever, both of which fall under the term legionellosis [1]. Among the more than 70 known species classified under the *Legionella* genus, the most common cause of legionellosis is *L. pneumophila*, which accounts for about 90% of laboratory-confirmed cases. Nevertheless, other non-pneumophila species, such as *L. bozemanae*, *L. dumoffii*, *L. micdadei*, *L. longbeachae*, and *L. gormanii*, are also causative agents of legionellosis [2,3].

The prerequisite for developing an infection is overcoming the bactericidal mechanisms of resident lung macrophages and establishing a niche that enables pathogen proliferation. Within alveolar macrophages, the bacteria remain surrounded by a membrane-bound vacuole, which protects them from lysosomal degradation [1]. The formation of a replicative niche known as the *Legionella*-containing vacuole (LCV) is attributed to numerous effector proteins that the bacteria introduce into the host cell and the lumen of the vacuole occupied by the pathogen. The transported proteins modulate the host cell’s life processes. The genomes of *Legionella* spp. encode numerous secretion systems, among which types II and IV are present in all studied *Legionella* species. The type I secretion system is limited to *L. pneumophila*, while the type VI secretion system is found in *L. cherrii*, *L. dumoffii*, and *L. gormanii* [4]. Effector proteins transported via these secretion systems manipulate the host cell’s life processes and enable the intracellular growth of bacteria. In the formation of the replication vacuole and the proper trafficking of effector proteins, glycerophospholipids (GPLs) play an important role, determining the physicochemical properties of membranes, including membrane disorder, fluidity, and permeability [5]. Lipidomic analysis of infected murine and human macrophages revealed that treatment with exogenous palmitoleic acid leads to reprogramming of the membrane phospholipid acyl chains and its disorder, thereby promoting the intracellular replication of *L. pneumophila* [6]. On the other hand, *Legionella* bacteria are capable of altering the proportions and composition of their glycerophospholipids depending on the culture conditions. One of the strategies used by *Legionella* bacteria for effective colonization of the human lungs is molecular mimicry, which involves the synthesis of phosphatidylcholine (PC). PC is the main phospholipid of the cell membrane in these bacteria and the dominant component of pulmonary surfactant. *Legionella* bacteria can synthesize PC through two independent pathways: the PE methylation (PMT) pathway and the PC synthase (PCS) pathway. The one-step synthesis PCS pathway, in which bacteria utilize extracellular choline, is more energetically favorable than the PMT pathway and allows bacteria to rapidly adjust their membrane physiology to adapt to changing environmental conditions. Thus, the bacteria produces enzymes that degrade lung surfactant, containing mostly PC, which serves as a substrate for phospholipase PlaB. The product of enzymatic degradation of PC is choline, which *Legionella* bacteria then use to synthesize their own PC.

GPLs are essential membrane-building lipids that play a crucial role in determining the physicochemical properties of biological lipid bilayers. Besides the mentioned PC, *Legionella* spp. comprise three other classes of phospholipids (PL)—phosphatidylethanolamine (PE), phosphatidylglycerol (PG), and cardiolipin (CL). The relative content of each class and their mutual proportions differ between various species. The *L. gormanii*, being the main subject of the conducted research, comprises 26% PC, 50% PE, 21% CL, and 3% PG [7]. It is also important to note that each class contains many different species depending on the fatty acid (FA) profile, i.e., 35 PE, over 13 PC, and more than 21 PG species were previously identified in *L. gormanii* [8,9]. However, these amounts and fatty acid profiles can be altered by exogenous factors, such as the above-mentioned choline. After choline supplementation of the growth medium, *L. gormanii* synthesizes 47% PC, 38% PE, 12% CL, and 3% PG [7]. This, in turn, influences interactions with host cells and antimicrobial peptides (AMPs). AMPs produced in the human body are increasingly recognized as potential agents in combating infections caused by *Legionella* spp. One of the peptides that can be potentially employed in killing the bacteria is the human cathelicidin LL-37. This helical peptide is characterized by a positive charge and is expressed, among others, in macrophages, epithelial cells, and neutrophils [10,11]. The LL-37 peptide participates in wound healing, cancer cell apoptosis, and angiogenesis promotion [12]. Moreover, it shows antimicrobial activity towards Gram-negative and Gram-positive bacteria by interacting with bacterial membranes [13].

Depending on the phospholipid structure and mutual proportions of these compounds, varying interactions are prominent in the membrane. In general, the phospholipids are striving for the maximization of van der Waals interactions between fatty acids and of the hydrogen bonds occurring between the hydrophilic headgroups [14]. However, the ability of certain components to interact with each other is determined by factors such as steric effects, the presence of H-bond donor and/or acceptor groups, and the length and degree of saturation of fatty acid chains. As a consequence, the differences in the spatial organization of multi-component membranes, their packing, ordering, molecular orientation, and stability occur depending on the membrane composition. These modifications heavily influence the peptide’s effectiveness towards bacterial membranes.

Determination of the antibiotics and other antimicrobial agents’ interactions with membranes of different compositions is of great importance for understanding the action mechanisms and designing new pharmaceuticals in the future [15]. This topic is the subject of numerous studies aimed at coherently describing the molecular processes occurring in natural conditions. The relations between compounds can be indirectly defined based on model membrane analyses. Many techniques, including Brewster angle microscopy, steady-state and time-resolved fluorescence methods, compression and adsorption isotherms’ registration, neutron or X-ray reflectivity, differential scanning calorimetry (DSC), or molecular dynamics (MD) simulations, are commonly used to investigate the model membranes properties and their changes under the antimicrobial agents’ influence [15,16,17,18,19,20,21,22]. These studies allow us to generally conclude what kind of interactions between components may occur in the analyzed system. Nevertheless, one of the most useful tools for quantifying the interactions occurring in a model membrane is a thermodynamic analysis based on the registered compression isotherms of individual phospholipids and their mixtures. The application of this approach involves determining the quantities such as the excess area ($A_{e}$), excess Gibbs energy (${\Delta G}_{e}$), and total Gibbs energy of mixing (${\Delta G}_{m}$). These calculations allow to obtain specific numerical data regarding the type and strength of interactions between the model membrane components in relation to the utterly miscible or immiscible ideal system. Additionally, as both the individual compounds and their mixtures are analyzed, the influence of each component on the membrane properties and their susceptibility to the peptide can be determined in more detail.

The aim of these studies was to primarily characterize interactions occurring in model membranes (Langmuir monolayers) created of phospholipids isolated from *L. gormanii* bacteria cultured on a medium with (PL + choline) and without (PL − choline) exogenous choline at 20 °C and 37 °C. The analysis of phospholipids isolated from bacteria both supplemented and not supplemented with choline allowed to determine the influence of physiologically adequate PL composition alterations on the membrane properties and antimicrobial agent effectivity. For this purpose, surface pressure-area (π−A) isotherms were determined for individual classes of phospholipids (PC, PE, PG, CL) and their mixtures (PL), which allowed us to calculate the compression modulus ($C_{S}^{-1}$), excess area ($A_{e}$), excess Gibbs energy (${\Delta G}_{e}$), and total Gibbs energy of mixing (${\Delta G}_{m}$). Then, analogous measurements and calculations were performed for the monolayers in the presence of the LL-37 peptide in the subphase to investigate the alterations in elasticity and strength of attractive or repelling forces between PL molecules caused by the LL-37 action.

Although studies of interactions in model bacterial membranes have been reported in the literature [23,24,25], they employed two or three compounds (standard phospholipids). The novelty of the presented research is that for the first time a multi-component mixture of phospholipids isolated directly from *L. gormanii* bacteria and separated into four classes were used to quantitatively determine the intermolecular interactions in a model membrane. The compression isotherms obtained for monolayers of individual classes of phospholipids played a reference role here, not selected single compounds as is usually used. We believe that this approach better illustrates the strength of interactions in real systems. Moreover, the thermodynamic analysis conducted for monolayers in the presence of the peptide allowed to quantitatively determine the LL-37 influence on the bacterial membranes at the molecular level. This brings us closer to developing the LL-37 antimicrobial action mechanism, which has not been clearly specified so far.

## 2. Results and Discussion

To provide a more detailed description of the LL-37 peptide effect on model bacterial membranes, it can be very helpful to determine the interactions in mixed monolayers of phospholipids derived from bacterial cells not supplemented or supplemented with choline, i.e., PL − choline and PL + choline monolayers, respectively. Thus, the π−A isotherms of individual classes were registered as a reference, and thermodynamic analysis using the excess functions for the PL monolayers in the absence or presence of the peptide in the subphase was conducted. Moreover, the monolayers’ elasticity (packing state) was characterized by the compression modulus values. We are aware that each class contains many different compounds [9], but for simplicity it was assumed that one class is one component of the phospholipid mixture (PL). Therefore, single-class monolayers are considered single-component, and multi-class monolayers are multi-component or mixed. The physicochemical description of single-and multi-class monolayers of phospholipids for *L. gormanii* can be found in our previous papers [9,19,26], but they do not include the thermodynamic analysis of interactions. Here, the interactions between phospholipid molecules of different classes are specified quantitatively, as they affect the overall susceptibility of the bacterial membrane to the action of the LL-37 peptide.

### 2.1. The π−A Isotherms and Compression Modulus $C_{S}^{-1}-\pi$ Dependencies

**Figure 1:**
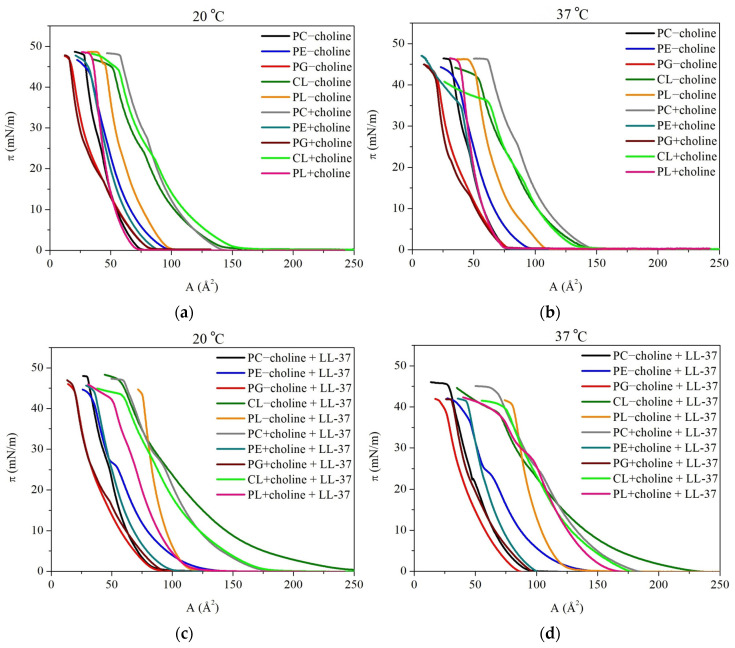
The π−A isotherms obtained for the individual phospholipid classes: PC, PE, PG, CL, and the PL mixtures isolated from *L. gormanii* bacteria cultured on a medium without (−choline) and with the addition of exogenous choline (+choline) at 20 °C and 37 °C, in the (**a**,**b**) absence or (**c**,**d**) presence of the LL-37 peptide.

In Figure 1, the surface pressure-area per molecule (π−A) isotherms obtained for the individual PC, PE, PG, and CL classes and the PL mixtures isolated from *L. gormanii* bacteria cultured on a medium without or with the addition of exogenous choline at 20 °C and 37 °C in the absence or presence of the LL-37 are shown. The model membranes were analyzed at two temperatures, i.e., 20 °C, referring to room temperature, is typically kept in studies on interfacial phenomena in Langmuir monolayers, enabling reference to literature data, whereas 37 °C corresponds to the physiological conditions for natural functioning of the LL-37 peptide while maintaining its high biological activity and thermal stability [27]. The temperature increase from 20 °C to 37 °C affects the LL-37 activity and allows to capture the influence of the packing state changes of phospholipid membrane on the peptide action, thereby peptide/phospholipid interactions considered below.

The π−A isotherms obtained for each model membrane were previously described [9,19,26]. Nevertheless, for a comprehensive overview, some crucial information will be mentioned here as well. As expected, each monolayer is characterized by slightly different properties resulting from differences in phospholipid composition that are induced by choline supplementation in the bacterial culture. Considering pure phospholipid monolayers (Figure 1a,b), it can be noted that the smallest mean molecular area values, at which the surface pressure increase begins, are observed for PG monolayers (78–83 ${Å}^{2}$) and the greatest for CL (144–161 ${Å}^{2}$), regardless of the experimental conditions. Moreover, both PG and CL, as well as PC isotherms, are characterized by inflection at surface pressure values of ~15 mN/m (PG) or 25–30 mN/m (CL and PC), similarly to PL − choline. The collapse pressure, meaning the surface pressure value at which the two-dimensional monolayer converts into the three-dimensional structures, is approximate in all analyzed models at 20 °C and is in a ~44–48 mN/m range. At 37 °C, a little decrease in this parameter can be observed, and the values are between 36 and 46 mN/m. It is also important to note that the collapse pressure values are greater for “+choline” monolayers, indicating higher stability. Comparing the “+choline” to the “−choline” single-component membranes’ mean molecular area values, the increase is noticed for PC monolayers (~89%), whereas PG and CL do not show significant changes in mean molecular area values and PE exhibits their decrease (by 9% at 20 °C and by 21% at 37 °C). Meanwhile, for PL + choline, the decrease in mean molecular area by 28–30% is observed at both temperatures as compared to PL − choline, pointing out the monolayer condensation. At 37 °C the monolayers’ expansion is more pronounced due to the greater thermal motion of the molecules, leading to an increase in the distance between them.

Furthermore, the LL-37 presence at the interface results in the π−A isotherm shift towards larger mean molecular area values in all analyzed monolayers (Figure 1c,d), indicating model membrane expansion and confirming the peptide–membrane interactions. The alterations in A values are equal on average: 22% for PC, 31% for PE, 10% for PG, and 36% for CL. The decrease in collapse pressure is also noted under the peptide influence, in a 0.2–10% range, with the smallest changes observed for PE and PC and the greatest alterations for CL. Considering the mixed model membranes, the biggest changes in monolayer behavior were observed for the PL + choline at 37 °C, with 110% molecular area value change and 14% collapse pressure decrease.

To determine the in-plane elasticity of the monolayers, which reflects their packing state, the compression modulus ($C_{S}^{-1}$) was calculated directly from the slope of the π−A isotherms, according to the formula [28]:

(1) $$C_{S}^{-1}=-A{\left(\frac{d\pi }{dA}\right)}_{T,p}$$

where T means the temperature and p is the atmospheric pressure.

**Figure 2:**
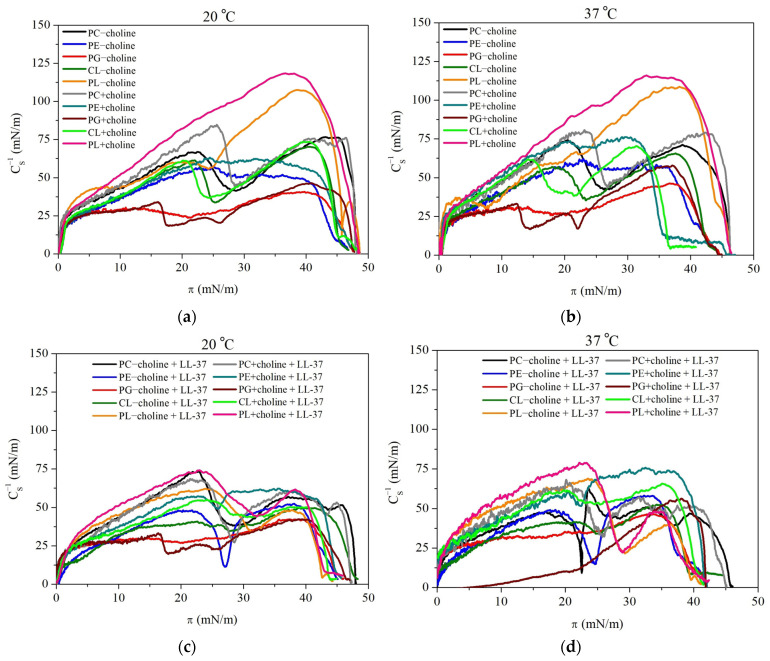
The $C_{S}^{-1}-\pi$ dependencies obtained for the individual phospholipid classes: PC, PE, PG, CL, and the PL mixtures isolated from *L. gormanii* bacteria cultured on a medium without (−choline) and with the addition of exogenous choline (+choline) at 20 °C and 37 °C, in the (**a**,**b**) absence or (**c**,**d**) presence of the LL-37 peptide.

The compression modulus describes the model membrane packing and provides information about the physical state of the monolayer at a specific compression stage (gas, liquid-expanded, liquid-condensed, or solid). The greater the $C_{S}^{-1}$ value, the less compressible (less elastic or more packed and ordered) the monolayer. The $C_{S}^{-1}$ values as a function of the surface pressure are presented in Figure 2.

Considering the monolayers in the absence of the LL-37 peptide (Figure 2a,b), the smallest values of compression modulus are observed for PG model membranes, where the $C_{S}^{-1}$ does not exceed 50 mN/m. This is indicative of the liquid-expanded (LE) state of the monolayer, according to the Davies and Rideal criterion [28]. For the remaining single-class model membranes, the values are in a range of 50–100 mN/m, pointing to the intermediate state between the LE and liquid-condensed (LC). It is also important to note that for PC, CL, and PG some minima on the $C_{S}^{-1}-\pi$ dependencies occur. This is in agreement with inflections observed on the π−A isotherms (Figure 1) and points to molecular reorganization within the monolayer at the interface, which can be associated with a second-order phase transition related to the changes in alkyl chain tilting. The greatest $C_{S}^{-1}$ of 107–119 mN/m is observed for the mixed PL monolayers, wherein the PL + choline is characterized by a greater degree of packing and ordering. The increased parameter values are noticeable for the single-class “+choline” monolayers as well in comparison to the “-choline” ones. Although the physical state of the model membranes is maintained, some changes in the degree of packing and ordering within the LE-LC phase occur. Considering the model membranes compressed to π around 35–45 mN/m, the $C_{S}^{-1}$ changes at 20 °C are equal to 16% for PE, 19% for PG, 5% for CL, and 10% for PL. PC at this temperature does not show a significant difference in $C_{S}^{-1}$ at these surface pressure values; however, at π~25 mN/m, before the monolayer reorganization, the PC + choline is characterized by 11% greater $C_{S}^{-1}$ in comparison to PC − choline. At 37 °C, the parameter alterations for PC, PE, PG, CL, and PL are equal to 11%, 29%, 26%, 8%, and 7%, respectively. Thus, it can be stated that the individual phospholipid classes isolated from bacteria supplemented with choline create model membranes characterized by lower elasticity. The temperature increase additionally enhances this effect. In the peptide presence, the monolayers show the maximal compression modulus values in a range of 40–74 mN/m at 20 °C and 39–79 mN/m at 37 °C (Figure 2c,d). Thus, in comparison to the model membranes compressed without the peptide addition, a compression modulus decrease is observed, which is in agreement with the π−A isotherm shift towards larger mean molecular areas (Figure 1). The LL-37 peptide seems to be the most effective towards “+choline” monolayers at 37 °C, indicating a synergistic effect of these two factors and a stronger disruptive action of LL-37 in relation to more ordered monolayers. The greatest changes in elasticity resulting from the peptide addition, amounting to 48%–63%, are noted for PL mixed model membranes. It is also crucial to mention that zwitterionic phospholipids (PC and PE), as well as the PL mixture in the presence of the LL-37, show a minimum at the $C_{S}^{-1}-\pi$ dependency at surface pressure around 25–30 mN/m. According to the literature, this surface pressure corresponds to the collapse pressure of the pure LL-37 monolayer [21,29]. Thus, it is likely that the excess peptide or peptide-rich phase is expulsed from the phospholipid model membrane into the subphase at this point. Contrary, in the case of anionic PG and CL, the mentioned minimum does not occur. As described in the previous paper [26], this is most probably the result of stable incorporation of cationic LL-37 into the anionic phospholipid model membranes and balancing of the negative charges.

Regarding the single-class monolayers, the fatty acid profile alterations are crucial for phospholipids’ organization changes under the choline supplementation influence, which are revealed in the π−A isotherm shifts and compression modulus changes. In general, the smaller mean molecular area values observed for the π−A isotherms (Figure 1) are a result of the saturated and/or long FA chains presence in the phospholipid structure, which promote greater packing because of stronger Lifshitz-van der Waals forces between the longer acyl chain molecules and translate to higher $C_{S}^{-1}$ (Figure 2) [30,31]. Meanwhile, the greater A values and lower compression modulus are a consequence of unsaturated, branched, or short FA in the molecules, causing steric effects [23,24,32]. Specifically, PE − choline contains more c17:0 in comparison to PE + choline, resulting in expansion, which is revealed by the greater mean molecular area and lower compression modulus. PG and CL classes do not show significant π−A isotherm shift; nevertheless, a small $C_{S}^{-1}$ increase is observed for “+choline” monolayers. As both of these classes are negatively charged, the repulsion between headgroups in single-compound monolayers may prevent closer packing of molecules, thus resulting in only slight alterations observed in mean molecular area and compression modulus values despite the FA composition changes [9]. PC model membranes show an unusual behavior considering the modifications evoked by choline presence. As mentioned before, for PC + choline, the π−A isotherms shift towards greater mean molecular values are noted, suggesting expansion; however, the compression modulus reveals lower elasticity of PC + choline in comparison to PC − choline. Based on the specific FA profile [9,26], it can be stated that the greater amount of long-chain acids (C19–C21) in the PC + choline model membrane provokes greater packing and ordering (Figure 2) due to the stronger Lifshitz van der Waals interactions. However, this monolayer also comprises more c17:0 and less saturated FA, which could favor the mean molecular area increase.

The PL − choline monolayer is characterized by the presence of greater amounts of unsaturated FA in comparison to PL + choline [26], which correlates well with the greater mean molecular area value and lower degree of packing and ordering of the mixed model membranes. These changes in fatty acid composition are important for the PL mixtures behavior as well; however, the proportions of classes and the headgroup interactions are crucial here. The PL + choline, containing greater amounts of PC (47%) and less negatively charged PG and CL (15%), shows greater condensation than the PL – choline, comprising 26% PC and 24% anionic lipids. The larger amounts of anionic compounds result in monolayer expansion due to the occurring repulsion between negative charges. Moreover, CL is increasing the membrane elasticity due to the four FA chains presence and the resulting steric effects [33,34]. Contrary to this, the increase in PC/PE ratio promotes a closer arrangement of phospholipids, as the PC molecule’s cylindrical shape favors condensation [35]. These phenomena are crucial for the interactions occurring between phospholipids in the presence of LL-37 and the model membrane susceptibility to the peptide.

### 2.2. Thermodynamic Analysis of Intramembrane Interactions

Based on the π−A isotherm data gained, the excess area ($A_{e}$), excess Gibbs energy (${\Delta G}_{e}$), and total Gibbs energy of mixing (${\Delta G}_{m}$) were determined to characterize the nature and magnitude of interactions between molecules as well as the thermodynamic stability of the PL − choline and PL + choline monolayers at defined surface pressures from 5 mN/m to 40 mN/m. Knowing the quantitative composition of the PL − choline and PL + choline mixtures [19], first the surface area occupied by PL molecules in an ideal monolayer ($A_{id}$) was calculated according to the formula presented as Equation (2) [36,37,38,39]:

(2) $$A_{id}=A_{1}X_{1}+A_{2}X_{2}+A_{3}X_{3}+A_{4}X_{4}$$

where $A_{1},A_{2},A_{3},and\;A_{4}$ refer to mean molecular areas at given surface pressure obtained from the PC, PE, PG, and CL isotherms; and $X_{1},X_{2},X_{3},and\;X_{4}$ are the molar fractions of individual phospholipid classes in the PL mixture.

The area calculated for ideal mixed monolayers was then compared to the actual area acquired for the quaternary monolayers ($A_{1234}$) providing the excess area ($A_{e}$) (Equation (3)). The values of this parameter allow to determine whether there are deviations related to stronger attractive or repulsive interactions in the multi-component (multi-class) PL monolayer in relation to an ideal system.

(3) $$A_{e}=A_{1234}-A_{id}$$

where $A_{1234}$ is the mean molecular area obtained for PL − choline and PL + choline monolayers at given surface pressure.

**Figure 3:**
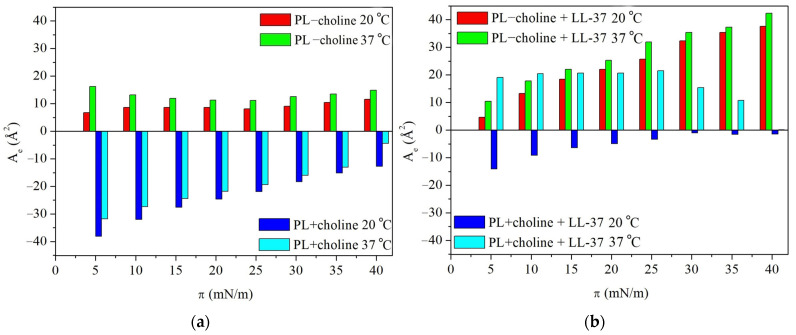
The excess area $A_{e}$, depending on the surface pressure π, obtained for the “−choline” and “+choline” multi-class monolayers at 20 °C and 37 °C, in the (**a**) absence or (**b**) presence of the LL-37 peptide.

The excess area characterizes the interactions between phospholipids in the multi-class monolayer qualitatively. If the parameter is equal to 0, the analyzed mixed monolayer exhibits the characteristics of an ideal system, as the area observed for the mixture is identical to the sum of the surface areas of the individual components (here classes), suggesting that the components are utterly immiscible or miscible. The negative $A_{e}$ values indicate the presence of stronger attractive forces, while the positive ones point to the increased repulsive interactions in comparison to the ideal monolayer [36,37,38,39]. The values of the excess area obtained at different surface pressures are presented in Figure 3.

Another parameter that describes quantitatively the interactions in complex monolayers is the above-mentioned excess Gibbs energy of mixing ($∆G_{e}$), determined on the basis of the excess area values at specific surface pressures (Equation (4)).

(4) $$∆G_{e}=N_{A}\int_{0}^{\pi }A_{e}d\pi$$

where $N_{A}$ is the Avogadro’s number.

**Figure 4:**
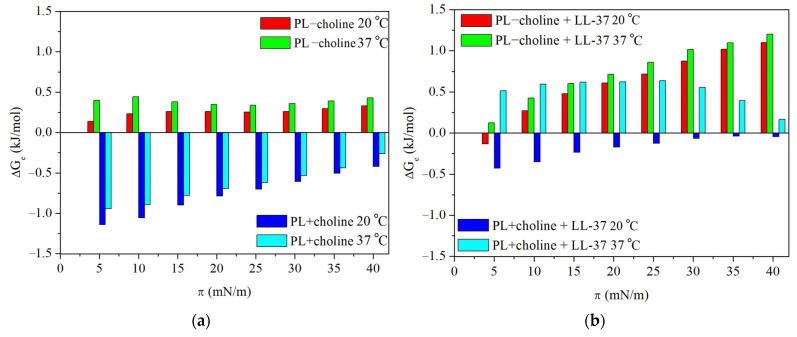
The excess Gibbs energy of mixing $∆G_{e}$, depending on the surface pressure π, obtained for the “−choline” and “+choline” multi-class monolayers at 20 °C and 37 °C, in the (**a**) absence or (**b**) presence of the LL-37 peptide.

Besides the type of interactions occurring between the PL molecules, $∆G_{e}$ allows one to estimate the strength of the repulsive or attractive forces [36,37,38,39]. The greater the deviation from the ideal mixed monolayer, the stronger the interactions of a specific type. The $∆G_{e}$ values obtained for the analyzed monolayers are shown in Figure 4.

Finally, to describe the monolayer stability, the total Gibbs energy of mixing ($∆G_{m}$) was calculated according to the formulas below (Equations (5) and (6)) [36,37,38,39].

(5) $${∆G}_{m}={∆G}_{e}+{∆G}_{id}$$

(6) $${∆G}_{id}=RT(X_{1}lnX_{1}+X_{2}lnX_{2}+X_{3}lnX_{3}+X_{4}lnX_{4})$$

where ${∆G}_{id}$ is the ideal Gibbs energy of mixing only related to entropy, R refers to the gas constant, and T is the temperature.

**Figure 5:**
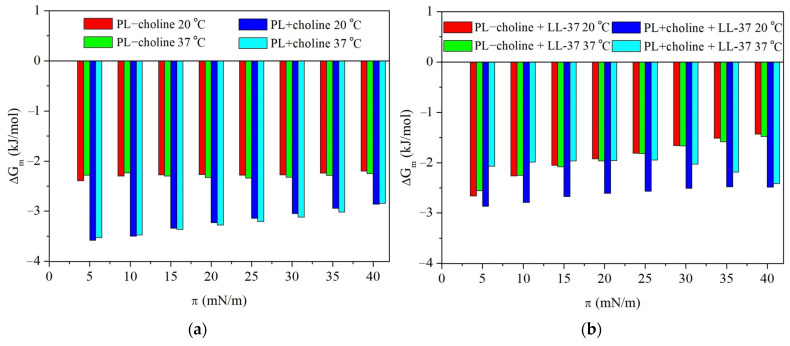
The total Gibbs energy of mixing $∆G_{m}$, depending on the surface pressure π, obtained for the “−choline” and “+choline” multi-class monolayers at 20 °C and 37 °C, in the (**a**) absence or (**b**) presence of the LL-37 peptide.

The sign of ${∆G}_{m}$ is considered a criterion of monolayer’s stability determination. The negative ${∆G}_{m}$ values are indicative of a thermodynamically more stable and favorable mixed state at any given surface pressure than the adequate unmixed one [40]. The total Gibbs energy of mixing for analyzed PL − choline and PL + choline monolayers is presented in Figure 5.

#### 2.2.1. Interactions in the Model Membranes in the Absence of the LL-37 Peptide

Without the LL-37 peptide, the PL − choline monolayer, both at 20 °C and 37 °C, is characterized by stronger repulsive forces ($A_{e}>0$) between the PL components than the single-class monolayers (Figure 3). Contrary, PL + choline shows vaster attraction ($A_{e}<0$) than an ideal mixing film. The magnitude of the interactions is reflected in ${∆G}_{e}$ values (Figure 4). They are in the range from −1141 J/mol to 428 J/mol, with negative values for PL + choline and positive ones for PL − choline. With the surface pressure increase during the compression, ${∆G}_{e}$ values are increasingly less negative for PL + choline, indicating the weaker attraction. Meanwhile, for PL – choline, a noticeable minimum appears at 25 mN/m, implying the slightest repulsion both at 20 °C and 37 °C.

It is also important to mention that the temperature increase provokes greater repulsive forces in the mixed monolayers revealed by the increased ${∆G}_{e}$ values (Figure 4). This is due to the higher kinetic energy of the molecules caused by the heat delivered to the system. Moreover, the temperature increase affects the hydrogen bonds forming between molecules, as the number of H bonds per molecule decreases at greater temperatures [41]. This dependency can be related to either an increase in repulsion (PL − choline) or a decrease in attractive forces (PL + choline). Furthermore, as can be seen in Figure 5, the values of total Gibbs energy of mixing are negative for all PL complex monolayers. Therefore, it can be stated that PL − choline and PL + choline model membranes are more thermodynamically stable than single-class monolayers, both at 20 °C and 37 °C. The PL + choline is characterized by greater stability, as the attractive interactions identified by $A_{e}$ and ${∆G}_{e}$ calculations are stronger than in PL − choline. Contrary, PL − choline, due to the stronger repulsive forces occurring between the monolayer components, shows less negative $∆G_{m}$ values, confirming smaller thermodynamic stability than that of PL + choline. For instance, at a pressure of 25 mN/m, the difference in $∆G_{m}$ values is about 863 J/mol. This is consistent with the greater mean molecular area values observed on the π−A isotherms (Figure 1a,b) registered for PL − choline and lower degree of packing (Figure 2a,b), demonstrating stronger repulsion between the components in comparison to the PL + choline.

**Figure 6:**
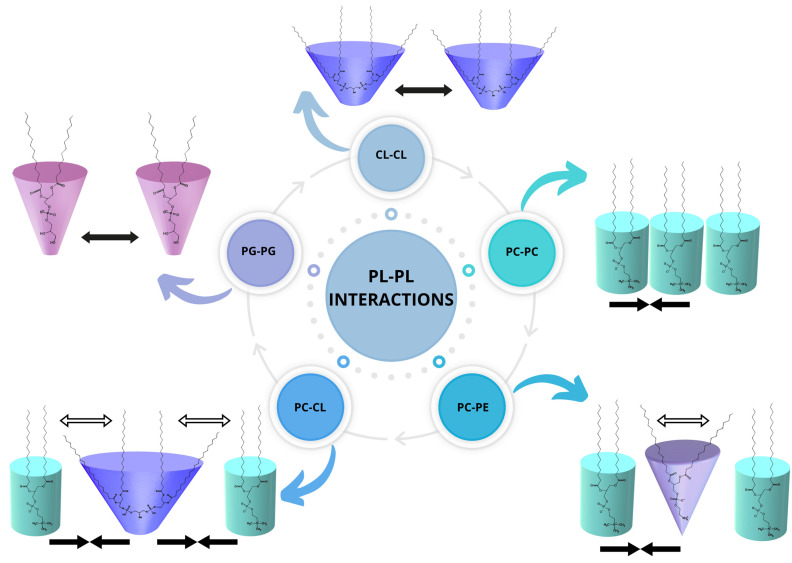
Possible types of interactions between the phospholipid (PL–PL) molecules in the *L. gormanii* model membranes, where PC—phosphatidylcholine (cyan cylinder); PE—phosphatidylethanolamine (purple cone); PG—phosphatidylglycerol (pink truncated cone); CL—cardiolipin (blue truncated cone); ↔ repulsion; →← attraction; ⇔ steric effects.

The above observations are in agreement with the physicochemical characteristics of the PL − choline and PL + choline monolayers, previously described in [9] and partially recalled in this paper. Repulsion in the PL − choline model membranes results in bigger mean molecular areas seen on the π−A isotherms (Figure 1), greater elasticity (Figure 2), and limited miscibility visible on the surface morphology images. On the contrary, the attraction between the phospholipid molecules in PL + choline is proved by smaller mean molecular areas, a higher degree of condensation, and better miscibility of the monolayer components [9]. To better understand the differences in the behavior of both types of monolayers, their quantitative composition and the specific PL–PL interactions affecting the mixed model membranes’ properties (Figure 6) should be considered.

The higher content of anionic phospholipids (24%) in the PL − choline monolayer [21] contributes to the increased density of negative charges in the model membrane, resulting in greater repulsion between molecules [42]. As the PG content is equal to only 3%, this phenomenon can mostly be attributed to the cardiolipin, consisting of the 21% of anionic lipids in PL − choline. In addition to the negative charge causing the cardiolipin molecules to repel each other, the steric effects during the monolayer compression can occur due to the CL molecule structure, i.e., the conical shape determined by a small headgroup and four fatty acid (FA) chains [33,34]. Thus, the increased amounts of CL can alter the interactions in the model membrane, resulting in weaker attraction or stronger repulsion between phospholipids. Not only CL but PE as well, due to the cone-shaped molecules, promote the phase separation, contributing to the increased repulsion in the membrane [43]. This kind of behavior is not necessarily noted in the single-class PE and CL model membranes (Figure 1 and Figure 2); however, it is important to mention that in the PL mixture, the headgroup interactions between four different classes occur, significantly altering the overall phospholipid behavior. This is a consequence of both hydrogen bond formation between many compounds and different charge distribution in the membrane, as opposed to all zwitterionic PE or all negatively charged CL monolayers. Taking these statements into account, it can be said that the PE (50%) and CL (21%) molecules determine the overall stronger repulsive interactions of lipids in PL − choline in comparison to the ideal system.

Meanwhile, the PL + choline model membrane exhibits stronger attractive interactions in comparison to both the ideal systems and PL − choline, which is revealed by the thermodynamic analysis (Figure 3, Figure 4 and Figure 5) as well as the π−A isotherms (Figure 1) and $C_{S}^{-1}-\pi$ dependencies (Figure 2). This phenomenon can be mostly associated with two factors. First of all, the greater amount of PC increases the attraction between phospholipids, as the cylindrical shape of the molecule promotes closer packing and ordering of the model membrane (Figure 6) [35,42]. Second of all, the decrease in CL (by 9%) and PE (by 12%) content results in weaker repulsion, as the steric effects described above are less prominent. These conclusions are in agreement with data available in the literature. It has been previously reported by Wilson et al. [20], who using molecular dynamics (MD) simulations for ternary lipid bilayer systems, found that the CL shows attractive interactions towards PC; however, for PE and PC/PE membranes, the cardiolipin addition results in greater repulsion and phase separation occurring between phospholipids [20]. Similar observations were made elsewhere by means of the Langmuir monolayer technique and Brewster angle microscopy for PE/PG model membranes, where the cardiolipin presence weakened the attractive interactions between components [33].

It is also important to mention the PG interactions with other phospholipids present in the PL − choline and PL + choline model membranes. The PG demonstrates the repulsive interactions with CL due to the fact that both of these phospholipid classes are negatively charged [44]. On the contrary, strong attraction takes place between PG and zwitterionic phospholipids, as was stated in the literature for PE/PG [44] and PC/PG systems [25]. However, as the analyzed *L. gormanii* comprises only 3% of PG and this percentage does not change under the influence of choline supplementation, these interactions are most likely not decisive for the quaternary model membrane behavior at the interface.

Furthermore, as mentioned before, the degree of packing of the model membrane, which is a direct result of the type and strength of interactions between phospholipids, is dependent on the FA chain profiles. The greater amount of unsaturated FA seems to contribute to the stronger repulsive forces in PL − choline, as in general, the presence of unsaturated chains results in stronger repulsive (or weaker attractive) interactions between phospholipids due to steric hindrance [23,24,32]. On the contrary, the greater content of saturated FA chains in PL + choline contributes to the stronger Lifshitz-van der Waals attraction between molecules in the monolayer (Figure 6).

#### 2.2.2. Interactions in the Model Membranes in the Presence of the LL-37 Peptide

In the presence of the peptide, the greater $A_{e}$ and $∆G_{e}$ values are observed for all monolayers compared to those without the LL-37 (Figure 3 and Figure 4). This is indicative of the strengthened repulsion between phospholipid molecules or the weakened attraction between them. The changes in magnitude of interactions in quaternary model membranes caused by the LL-37 peptide presence are quantitatively presented in Table 1. As the attractive or repulsive forces can be associated with degree of packing and ordering of the monolayers, the compression modulus ($C_{S}^{-1}$) values and their changes are also included below for comparison purposes. The $∆G_{e}$ and $C_{S}^{-1}$ alterations resulting from the peptide presence are considered at 25 mN/m, due to the fact that the pure LL-37 monolayer collapses at π above that value, and it can be desorbed towards the subphase [21,29].

Considering PL − choline, the $∆G_{e}$ increase is equal to 465.2 J/mol and 522.5 J/mol at 20 °C and 37 °C, respectively. For PL + choline at 37 °C, the $∆G_{e}$ value changes by 1255.8 J/mol, and interestingly, a change in the sign of the values from negative to positive points out a conversion of the interactions nature from attractive to repulsive (Figure 3 and Figure 4). Only in PL + choline at 20 °C do the interactions still show an attractive character ($A_{e}<0$), but are much weaker (575.2 J/mol $∆G_{e}$ increase). The above observations clearly demonstrate the significant contribution of the peptide to modifying the interactions between phospholipid molecules. It is also important to note that the repulsion increase or attraction forces decrease correlates well with the monolayer elasticity (degree of packing and ordering) alterations, revealed by the $C_{S}^{-1}$ changes (Table 1, Figure 2) and the mean molecular area increase on the π−A isotherms (Figure 1). The greatest effect is noticed for PL + choline at 37 °C (Table 1), proving the strongest interactions between this monolayer and the LL-37 peptide, which leads to the greatest membrane destabilization. These observations further confirm the synergistic influence of temperature increase and choline supplementation of the bacteria culture on the model membrane susceptibility to the LL-37. When the LL-37 peptide is added to the subphase, the less negative $∆G_{m}$ values are obtained over the full surface pressure range for all analyzed monolayers (Figure 5). They suggest smaller thermodynamic stability of the model membranes in comparison to that in the absence of the LL-37. These results further confirm the peptide’s antimicrobial activity by reducing monolayer stability.

The observed alterations in the type and strength of interactions between phospholipids are naturally a consequence of different affinity of the LL-37 to four phospholipid classes in the monolayer. As described previously, for anionic model membranes (CL and PG) during the compression with the LL-37, the stable peptide incorporation was noted, while in the PE and PC single-class monolayers the excess peptide or peptide-rich phase expulsion can take place at π above 25 mN/m [26]. This suggests low PE and PC affinity to the peptide, which is in agreement with experimental and MD simulation studies conducted by other researchers [21,29,45] that showed the LL-37 ability to interact preferentially with anionic PG model membranes due to the strong attractive forces between opposite charges favoring the parallel arrangement of LL-37 to the membrane plane.

**Figure 7:**
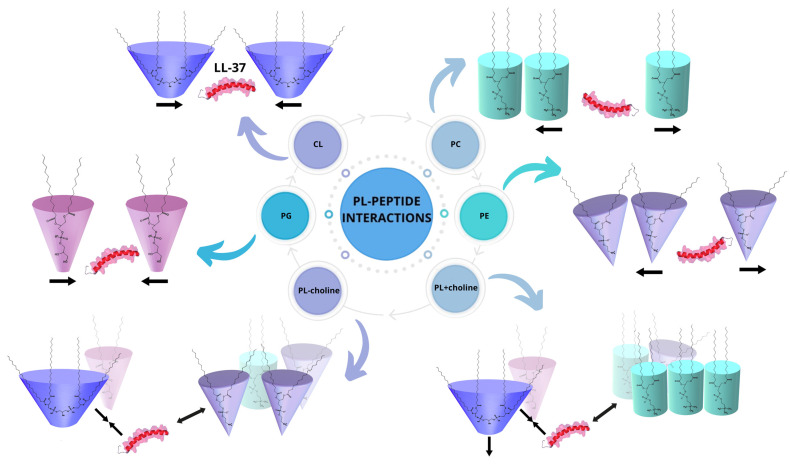
Possible types of interactions between the phospholipid and peptide (PL-PEPTIDE) molecules in the *L. gormanii* model membranes, where PC—phosphatidylcholine (cyan cylinder); PE—phosphatidylethanolamine (purple cone); PG—phosphatidylglycerol (pink truncated cone); CL—cardiolipin (blue truncated cone); LL-37—cathelicidin (red-pink molecule); ↔ repulsion; →← attraction.

As mentioned above, for PL − choline model membranes, containing 50% PE and 26% PC, the increase in repulsion is observed during the compression. Moreover, at surface pressures higher than 25 mN/m, even stronger repulsion takes place, suggesting that the LL-37 molecules still remain embedded in the model membrane despite being repelled by PC/PE molecules. This may be due to the 21% CL content, which, as anionic phospholipid, strongly attracts the positively charged peptide [46,47] and prevents its expulsion. Therefore, the observed intermolecular interactions in the mixed PL − choline monolayer could be assigned to the combined effects of dominating repulsion between the LL-37 and zwitterionic lipids and the peptide’s attraction towards anionic CL and PG. These PL-LL-37 interactions are schematically presented in Figure 7.

Considering the PL + choline model membranes containing 47% PC and 38% PE, at 20 °C the significant decrease in attraction indicates that the interactions between the LL-37 and increased amounts of zwitterionic lipids are prominent for the observed repulsive forces (Figure 7). At 37 °C, this effect is much stronger, and unlike in other monolayers, the repulsion forces decrease at surface pressure above 25 mN/m, which can be correlated with the expulsion of the peptide or peptide-rich phase from the monolayer. Moreover, the decreased amounts of anionic lipids in PL + choline contribute to the smaller attraction of the peptide towards the model membrane, which facilitates the LL-37 expulsion to the subphase at 37 °C. It is also important to mention that PL + choline is more susceptible to the LL-37 in comparison to PL − choline, despite the fact that PL + choline is more ordered (Figure 2) and thus would seem more resistant. This is due to the fact that the peptide addition results in greater destabilization and noted changes in a more packed model membrane than in the monolayer characterized by a larger disorder, where the peptide can be accommodated and interact with phospholipids without causing significant additional effects [48]. Sevcsik et al. [21] using differential scanning calorimetry (DSC) for a dipalmitoyl-PE/dipalmitoyl-PG (DPPE/DPPG) mixture showed that the cationic peptide LL-37 preferentially interacts with the negatively charged DPPG to form peptide-enriched PG domains, thereby depleting the mixture in this phospholipid. In another study, DSC themograms obtained for dimyristoyl-PC (DMPC) vesicles revealed the formation of peptide-rich domains by a lipid-induced peptide aggregation process [49]. This scenario supports our interpretation that the peptide phase containing anionic CL and/or PG desorbs from the monolayer, which then becomes enriched in zwitterionic PC/PE phospholipids. This process leads to large disruptions in the structure of the model membrane that are essential in the context of the general antibacterial action mechanism. As demonstrated above, the membrane-disruptive activity of LL-37 is affected not only by the charges but also by other features, including the packing density, H-bond formation, and molecular shape, consistent with the studies of Sevcsik et al. [21].

## 3. Materials and Methods

*L. gormanii* (ATCC 33297) bacteria were cultured on buffered charcoal yeast extract (BCYE) agar plates (Oxoid, Basingstoke, UK) with or without the 100 µg/mL choline chloride (Sigma-Aldrich, St. Louis, MO, USA) supplementation. After 3 days at 37 °C, 5% CO_2_, the biomass was collected. Phospholipids (PLs) were then isolated from bacteria by means of the Bligh and Dyer procedure [50], employing the chloroform (Avantor Performance Materials Poland S.A., Gliwice, Poland; purity > 99.9%) and methanol (ROMIL Chemicals Ltd., Cambridge, UK; purity > 99.9%) mixture (1/2, *v*/*v*). In order to purify and separate the mixture into individual classes, the extracted phospholipids were subjected to one-dimensional thin-layer chromatography (TLC) using the 10 cm × 10 cm silica gel 60 F254 plates (Merck, Darmstadt, Germany). To remove pigments and contaminants, ~2 mg of phospholipids dissolved in a chloroform/methanol mixture (4/1, *v*/*v*) were applied to the silica plate and developed in a chloroform/methanol/acetic acid mixture (98/2/1, *v*/*v*/*v*). Then, the PLs were identified in iodine vapor, scrapped off of the plate, and separated from the silica using 3 mL of chloroform/methanol (1/1, *v*/*v*) solvents. Next, the phospholipids were dried under nitrogen gas, weighted, and stored at −20 °C. To separate the phospholipids into individual classes (phosphatidylcholine, PC; phosphatidylethanolamine, PE; phosphatidylglycerol, PG; cardiolipin, CL), the PLs were developed in a chloroform/methanol/glacial acetic acid (13/5/2, *v*/*v*/*v*) mixture. The remaining steps were carried out as before.

The Langmuir–Blodgett trough (KSV 2000 Standard, KSV Instruments, Helsinki, Finland) equipped with symmetrical barriers and a Wilhelmy plate balance (0.1 mN/m accuracy) was employed to determine the surface pressure as a function of the surface area per molecule (π−A compression isotherms) at 20 °C and 37 °C. To keep the temperature constant, an external water circulating system (Lauda, Schwechat, Austria) was utilized. The obtained *L. gormanii* phospholipids were dissolved in chloroform (Avantor Performance Materials Poland S.A., Gliwice, Poland; purity > 99.9%) and methanol (ROMIL Chemicals Ltd., Cambridge, UK; purity > 99.9%) at a volume ratio of 4/1 to obtain 1 mg/mL solutions and applied to the subphase surface (0.01% acetic acid) using a Hamilton microsyringe. After 10 min of solvent evaporation, the monolayer compression was conducted with the symmetric barriers at 10 mm/min speed, and the π−A isotherms were registered. The measurements with the LL-37 peptide (LL-37 (human) trifluoroacetate salt, Merck, Darmstadt, Germany; purity ≥ 95%) were carried out by applying 10 µL of the peptide solution (1 mg/mL) to the subphase surface (0.08 µg/mL in the bulk phase), leaving it for 2 h, and then dropping the PL solution and conducting the π−A isotherm registration analogously.

## 4. Conclusions

Interactions occurring in the multi-component monolayers of bacterial phospholipids and their stability at the liquid/air interface with and without the LL-37 addition into the subphase were investigated by the Langmuir monolayer technique. The π−A compression isotherms acquired at 20 °C and 37 °C allowed to determine the compression modulus ($C_{S}^{-1}$), as well as the excess area ($A_{e}$), excess (${\Delta G}_{e}$), and total (${\Delta G}_{m}$) Gibbs energy of mixing, providing information on the type and magnitude of interactions. The conducted analyses showed a significant change in interactions between phospholipids depending on the components mutual proportions in the model membrane. Moreover, it was revealed that the LL-37 presence affected the attractive/repulsive forces to a different extent in PL − choline and PL + choline monolayers, which confirmed the composition-dependent membrane-lytic action of the peptide.

It was found that in PL − choline model membranes there are stronger repulsive interactions between components due to the greater CL and unsaturated FA content, resulting in limited miscibility of components. Meanwhile, in PL + choline, the attractive forces are more dominant as a result of increased PC content, a reduction in anionic phospholipid percentage, and greater amounts of saturated fatty acids. Consequently, this model membrane is characterized by better miscibility. Such a molecular organization seems more susceptible to the LL-37 action, which is indicated by the largest alterations in the thermodynamic quantities determined for PL + choline. This is due to the greater repulsion between the LL-37 and zwitterionic phospholipids dominant in the PL + choline composition, leading to substantial destabilization. The considerable decrease in the magnitude of interactions occurring in the PL + choline monolayer and its overall thermodynamic stability in the presence of the LL-37 demonstrate the great peptide potential in terms of antibacterial activity. The presented studies give a better understanding of the behavior of bacterial membrane properties affected by the LL-37 peptide on a molecular level. Broadening the knowledge on this subject can contribute to the development of new pharmaceuticals effective against infections caused by *Legionella* bacteria.

## Figures and Tables

**Table 1:** The values of $∆G_{e}$ and $C_{S}^{-1}$ at π = 25 mN/m obtained for PL − choline and PL + choline monolayers at 20 °C and 37 °C and the parameters’ alterations caused by the LL-37 peptide addition.

	$∆G_{e}$ (kJ/mol)	$∆G_{e}$ Difference (kJ/mol)	$C_{S}^{-1}$ (mN/m) [19]	$C_{S}^{-1}$ Difference (mN/m)
PL − choline 20 °C	0.253	0.465	56.6	4.2
PL − choline + LL-37 20 °C	0.718		60.8	
PL − choline 37 °C	0.338	0.522	75.8	11.7
PL − choline + LL-37 37 °C	0.861		64.1	
PL + choline 20 °C	−0.700	0.575	94.9	21.4
PL + choline + LL-37 20 °C	−0.125		73.5	
PL + choline 37 °C	−0.620	1.255	99.4	32.0
PL + choline + LL-37 37 °C	0.635		67.4	

## Data Availability

Data are contained within the article.
